# Case–control study of prolactin and placental lactogen in SGA pregnancies

**DOI:** 10.1530/RAF-21-0020

**Published:** 2021-09-10

**Authors:** Sharon R Ladyman, Caroline M Larsen, Rennae S Taylor, David R Grattan, Lesley M E McCowan

**Affiliations:** 1 Centre for Neuroendocrinology and Department of Anatomy, School of Biomedical Sciences, University of Otago, Dunedin, New Zealand; 2 Maurice Wilkins Centre for Molecular Biodiscovery, Auckland, New Zealand; 3 Department of Obstetrics and Gynaecology, Faculty of Medical and Health Science, University of Auckland, Auckland, New Zealand

**Keywords:** small for gestational age, prolactin, human placental lactogen, pregnancy

## Abstract

Prolactin and placental lactogens increase during pregnancy and are involved with many aspects of maternal metabolic adaptation to pregnancy, likely to impact on fetal growth. The aim of this study was to determine whether maternal plasma prolactin or placental lactogen concentrations at 20 weeks of gestation were associated with later birth of small-for-gestational-age babies (SGA). In a nested case–control study, prolactin and placental lactogen in plasma samples obtained at 20 weeks of gestation were compared between 40 women who gave birth to SGA babies and 40 women with uncomplicated pregnancies and size appropriate-for-gestation-age (AGA) babies. Samples were collected as part of the 'screening of pregnancy endpoints' (SCOPE) prospective cohort study. SGA was defined as birthweight <10th customized birthweight centile (adjusted for maternal weight, height, ethnicity, parity, infant sex, and gestation age) in mothers who remained normotensive. No significant differences were observed in concentrations of prolactin or placental lactogen from women who gave birth to SGA babies compared with women with uncomplicated pregnancies. However, a sex-specific association was observed in SGA pregnancies, whereby lower maternal prolactin concentration at 20 weeks of gestation was observed in SGA pregnancies that were carrying a male fetus (132.0 ± 46.7 ng/mL vs 103.5 ± 38.3 ng/mL, mean ± s.d., *P* = 0.036 Student’s *t*-test) compared to control pregnancies carrying a male fetus. Despite the implications of these lactogenic hormones in maternal metabolism, single measurements of either prolactin or placental lactogen at 20 weeks of gestation are unlikely to be useful biomarkers for SGA pregnancies.

**Lay summary:**

Early identification during pregnancy of small for gestational age (SGA) babies would enable interventions to lower risk of complications around birth (perinatal), but current detection rates of these at risk babies is low. Pregnancy hormones, prolactin and placental lactogen, are involved in metabolic changes that are required for the mother to support optimal growth and development of her offspring during pregnancy. The levels of these hormones may provide a measurable indicator (biomarker) to help identify these at risk pregnancies. Levels of these hormones were measured in samples from week 20 of gestation from women who went on to have SGA babies and control pregnancies where babies were born at a size appropriate for gestation age. Despite the implications of prolactin and placental lactogen in maternal metabolism, no significant differences were detected suggesting that single measures of either prolactin or placental lactogen at 20 weeks gestation are unlikely to be useful biomarker to help detect SGA pregnancies.

## Introduction

Small for gestational age (SGA) infants account for about 30–50% of non-anomalous still-born infants and those that survive have an increase in the risk for neurodevelopmental delays, and cerebral palsy (Baschat 2011, PMMRC 2018). Furthermore, the effects of being born SGA impacts on health as an adult, with increased risk of cardiovascular complications and diabetes in later life (Barker *et al.* 2007). Identification during pregnancy, leading to intervention and timely delivery has been reported to lead to a four-fold reduction in perinatal death and severe asphyxia (Lindqvist & Molin 2005). Identifying SGA before birth is difficult, however, and using population-based growth charts less than a quarter of all SGA babies are identified before birth (Wright *et al.* 2006). Using customized antenatal growth charts that take into account a range of factors including maternal weight, height, and ethnicity can improve antenatal identification of SGA infants, but even with this improvement detection rates are reported to be only around 50% (Wright *et al.* 2006, Roex *et al.* 2012). Recent analysis of a multi-centre cohort study (SCOPE) has identified key clinical variables at 15 weeks that are associated with later development of SGA (McCowan *et al.* 2013). In that study, only one quarter (24.5%) of all SGA infants were identified before birth, highlighting the need to have improved methods for detecting SGA during pregnancy (McCowan *et al.* 2013). As indicated in  McCowan *et al*. (2013), the key next step for the development of a personalized algorithm for prediction of SGA is the identification of reliable biomarkers that can be combined with clinical risk factors (McCowan *et al.* 2013).

One of the key hormones associated with pregnancy is prolactin. While prolactin is thought of primarily as a lactation hormone (Trott *et al.* 2012), it has also been implicated in a wide range of other functions (Bole-Feysot *et al.* 1998, Grattan 2015), particularly during pregnancy (Grattan & Le Tissier 2015). Prolactin concentrations increase progressively throughout pregnancy in women (Tyson *et al.* 1972, Grattan 2001, Romero *et al.* 2017, Aghaeepour *et al.* 2018) and the placenta also contributes the closely related human placental lactogen (hPL (CSH1), or chorionic somatomammotropin) as an additional source of circulating hormone that can activate the prolactin receptor. Human placental lactogen is secreted from the syncytiotrophoblast starting at 6 weeks of gestation and then increasing to extremely high levels as gestation advances (Braunstein *et al.* 1980). Indeed, recent proteomic studies of human pregnancy blood samples have identified prolactin in the top 1% of proteins showing increased expression during pregnancy (Romero *et al.* 2017, Aghaeepour *et al.* 2018), with hPL also as one of the highest induced proteins (Aghaeepour *et al.* 2018). Prolactin and hPL play an important role in mediating the maternal metabolic adaptations that help to establish a positive energy balance to meet the demands of fetal growth and also to prepare for subsequent demands of lactation (Augustine *et al.* 2008, Grattan & Kokay 2008, Newbern & Freemark 2011, Grattan & Le Tissier 2015). These adaptations ensure appropriate glucose and amino acid availability to the fetus (Handwerger 1991). Prolactin may also have a more direct role in fetal growth by influencing trophoblast invasion in early pregnancy (Stefanoska *et al.* 2013), which when impaired, has been associated with SGA pregnancies (Knofler 2010). In animal models, experimental suppression of placental lactogenic hormones is associated with fetal growth restriction (Lee *et al.* 2015). Reduced expression of genes for *hPL* (*CSH1*) and the placental growth hormone variant (*GH*-V) have been observed in terms of placentas associated with SGA newborns (Mannik *et al.* 2010), suggesting that secretion of these placental hormones may be compromised when fetal growth is impaired. Changes in expression of imprinted genes that control *hPL* expression (John 2013) have also been associated with changes in fetal growth (Jensen *et al.* 2014, Janssen *et al.* 2016). Similarly, animal models involving manipulation of these placental regulatory genes also result in changes in fetal growth (Tunster *et al.* 2016). These data highlight the potential for placental hormones to serve as biomarkers of impaired fetal growth. Collectively, these data are consistent with the hypothesis that changing levels of circulating hormones that act through the prolactin receptor, including prolactin and growth hormone from the maternal pituitary (and also decidua), and hPL (and to a lesser extent GH-V) from the placenta, impact on fetal growth and could potentially serve as biomarkers of impaired fetal growth. In our previous work, we showed that while circulating GH-V correlated with fetal growth in large for gestational age babies, there was no detectable deficit in SGA pregnancies (Liao *et al.* 2016). The aim of the current study was to investigate if hPL or pituitary-derived prolactin levels were reduced at 20 weeks of gestation in pregnancies that resulted in birth of a SGA baby.

## Methods

### Study design

The participants were healthy, nulliparous women with singleton pregnancies who were recruited in Auckland, New Zealand, to be included in the multi center 'screening of pregnancy endpoints' (SCOPE) study. The SCOPE study was a prospective, multicentre international screening study which aimed to develop screening tests to predict preeclampsia, SGA infants, and spontaneous preterm births. Ethics approval and consent to participate was obtained from local ethics committees, including New Zealand (AKX/02/00/364) and all women provided written informed consent. Detailed methods of the SCOPE study are described elsewhere (McCowan *et al.* 2010). For the current study, 40 case and 40 control samples were randomly selected from the participants who were recruited in Auckland, New Zealand, (total of 1296 uncomplicated AGA pregnancies and 159 normotensive SGA pregnancies) and who had a 20-week plasma specimen.

For the current investigation, plasma samples obtained at 20 weeks of gestation were used. Following birth (usually within 72 h of giving birth), pregnancy outcome data and infant measurements were recorded by research midwives. An SGA outcome was conventionally defined as a birthweight of less than the tenth customised centile, adjusted for maternal height, booking weight, ethnicity, sex of infant, and gestation at birth. The control group in this nested case–control study comprised of 40 women with singleton, uncomplicated pregnancies who delivered babies with birthweight >10th customised centile, while the SGA group comprised 40 women with singleton, normotensive pregnancies who delivered SGA babies. The number of 40 per group was determined based on power analysis (for an effect size of 0.8) based on unforeseen, preliminary data obtained from another study using samples from the SCOPE study.

### Assays

Prolactin and placental lactogen concentrations were measured in duplicate in 20 weeks of gestation maternal plasma samples using commercially available ELISA assays (human prolactin ELISA (25-PROHU-E01) and human placental lactogen ELISA (20-HPLHU-E01) both from Alpco Diagnostics). Assays were performed as described in the manufacturer’s instructions. For the hPL assay, the intraassay CV was 2.8% and interassay CV was 13.2%. For the prolactin assay, the intraassay CV was 3.2% and the interassay CV was 5.1%. For each assay plate, both control and case studies were included.

### Statistical analysis

All hormone data was analyzed for normality using the D’Agostino and Pearson normality test. When data was normally distributed, a Student’s *t*-test was used to assess the significance between groups, whereas when data was not normally distributed, a Mann–Whitney nonparametric test was used. The Mann–Whitney test was used for human placental lactogen analysis of all combined samples and samples from mothers carrying female fetuses; and a two-tail Student’s *t*-test was used for the prolactin analysis along with analysis for human placental lactogen in samples from mothers carrying male fetuses. Since the hypothesis was that low lactogenic hormone activity would be associated with SGA pregnancies, we also analyzed the data using a one-tail test. Previous work has suggested fetal sex-specific association of low hPL and reduced fetal growth (Lagerstrom *et al.* 1990), therefore, the data was also analyzed based on fetal sex. Each group was assessed for outliers using the ROUT outlier test. Only one sample from the control group of the hPL data was removed due to testing positive as an outlier (extremely high at 13.43 ng/mL), however, the outcome of the statistical analysis did not differ with or without the inclusion of this value (with: *P* = 0.7736, without: *P* = 0.7736 using two-tailed Mann–Whitney tests). Statistical differences in the study population characteristics between control pregnancies and SGA pregnancies were analyzed with Student’s *t*-test for continuous variables and with chi-square tests for categorical variables. For hormone data, all values were expressed as mean ± s.d., and for the study population characteristics, continuous variables are expressed as mean (s.d.) while categorical variables are expressed as numbers (percentage).

## Results

Maternal characteristics and pregnancy outcomes for the normotensive SGA and uncomplicated pregnancy groups are detailed in Table 1. There were no differences between groups for any maternal characteristics. SGA cases were delivered earlier than controls (*P* = 0.03).

**Table 1:** Study population characteristics at 15 weeks and pregnancy outcomes. Results expressed as *n* (%) or mean (s.d.). Continuous variables: *t*-test; categorical variables: chi-square or Fisher’s exact test.

	Normotensive SGA (*n* = 40)	Uncomplicated pregnancy (*n* = 40)	*P* value
Maternal details at 15 weeks			
Maternal age (y)	31.6 ± 4.4	31.3 ± 4.4	0.78
Caucasian ethnicity	37 (93%)	37 (93%)	1.0
Primigravid	29 (73%)	29 (73%)	1.0
Single	2 (5%)	0	0.49
No paid employment	0	3 (7.5%)	0.24
Socioeconomic index	51 ± 12	48 ±12	0.29
Smoker	1 (2.5%)	1 (2.5%)	1.0
BMI category (kg/m^2^)			0.58
<24.9	26 (65%)	25 (62.5%)	
25–29.9	10 (25%)	13 (32.5%)	
≥30.0	4 (10%)	2 (5%)	
Systolic BP (mmHg)	106 ± 10	107 ± 9	0.82
Diastolic BP (mmHg)	63 ± 9	66 ± 8	0.19
15-week extreme exercise (yes)	1 (2.5%)	0	1.0
Gestation at sampling (wks)	19.9 ± 0.6	19.9 ± 0.7	0.86
Pregnancy outcomes			
Birthweight (g)	2710 ± 398	3506± 353	<0.001
Sex of baby (girl)	18 (45%)	20 (50%)	0.82
Gestational age at delivery (weeks)	39.2 ± 2.4	40.2 ± 1.1	0.03
Customised birthweight centile	5.7± 2.6	45.9 ±23.7	<0.001
Preterm births (<37 weeks)	6 (15%)	0	0.08
Neonatal unit admission	8 (20%)	2 (5%)	0.09
Specimen analyzes at 20 weeks*			
Prolactin (ng/mL)	112.6 ± 44.9	128.2 ± 43.2	0.058 (0.116)
Human placental lactogen (mg/mL)	2.46 ± 0.74	2.64 ±1.84	0.464 (0.774)

*Specimen analyzes are expressed as mean (s.e.m.) and *P* values for both one-tailed and two-tailed statistical testing is given, with the latter being in brackets.

**Figure 1:**
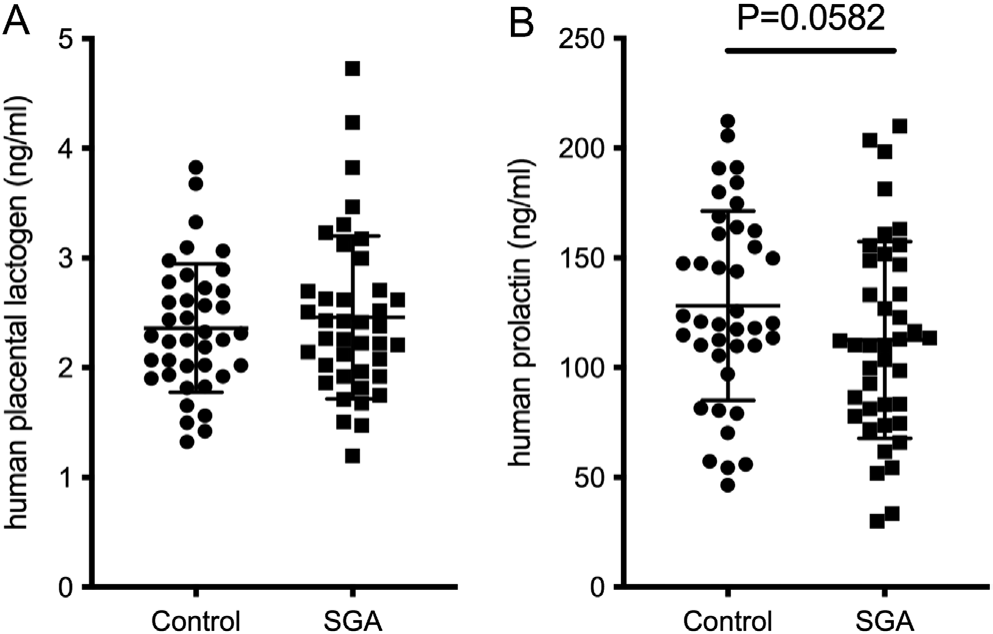
Placental lactogen (A) and prolactin concentrations (B) in maternal plasma samples collected from pregnant women at 20 weeks of gestation who went on to have babies of normal birth weight (control, *n*  = 40) or small for gestational age (SGA, <10th centile, *n*  = 40). One sample was removed from the control placental lactogen group as it was tested to be an outlier. Placental lactogen, *P* = 0.3868 (one-tailed Mann–Whitney nonparametric test as data were not normally distributed (SGA group *P* < 0.05 D’Agostino and Pearson normality test)). Human prolactin, *P* = 0.0582 (Student’s one-tailed *t*-test). Data shown as mean ± s.d.

**Figure 2:**
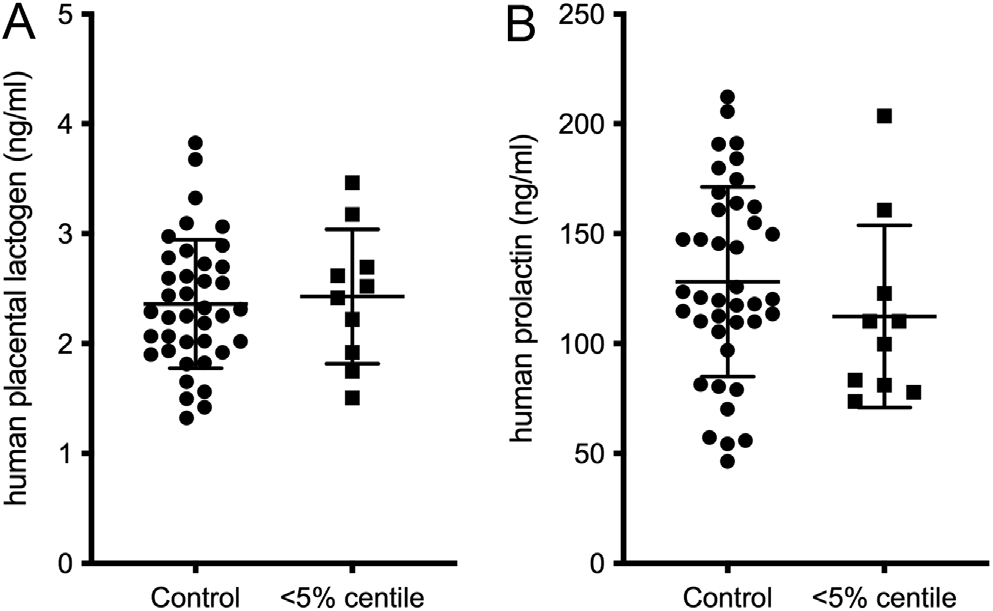
Placental lactogen and prolactin concentrations in maternal plasma samples collected from pregnant women at 20 weeks of gestation who went on to have babies of normal birth weight (control, *n*  = 40) or SGA (<5thcentile, *n*  = 10). One sample was removed from the control placental lactogen group as it was tested to be an outlier. Human placental lactogen *P* = 0.75, Student’s *t*-test. Human prolactin *P* = 0.30, Student’s *t*-test. Data shown as mean ± s.d.

No significant differences were detected in maternal plasma placental lactogen or prolactin concentrations between the uncomplicated pregnancy and SGA groups (placental lactogen: *P* = 0.7736 two-tailed Mann–Whitney tests, prolactin: *P* = 0.1164 two-tailed *t*-test) (Fig. 1). When specifically investigating the hypothesis that low lactogenic hormone activity would be associated with SGA pregnancies, there was no statistically significant difference in prolactin concentrations in pregnancies that went on to have a SGA infant compared to those with uncomplicated pregnancies (*P* = 0.058). To determine whether any trend might become more pronounced with more severe SGA, the data was further examined using only the samples from more extreme SGA cases (<5th centile). No significant differences were found between control and SGA groups in this further analysis (Fig. 2).

**Figure 3:**
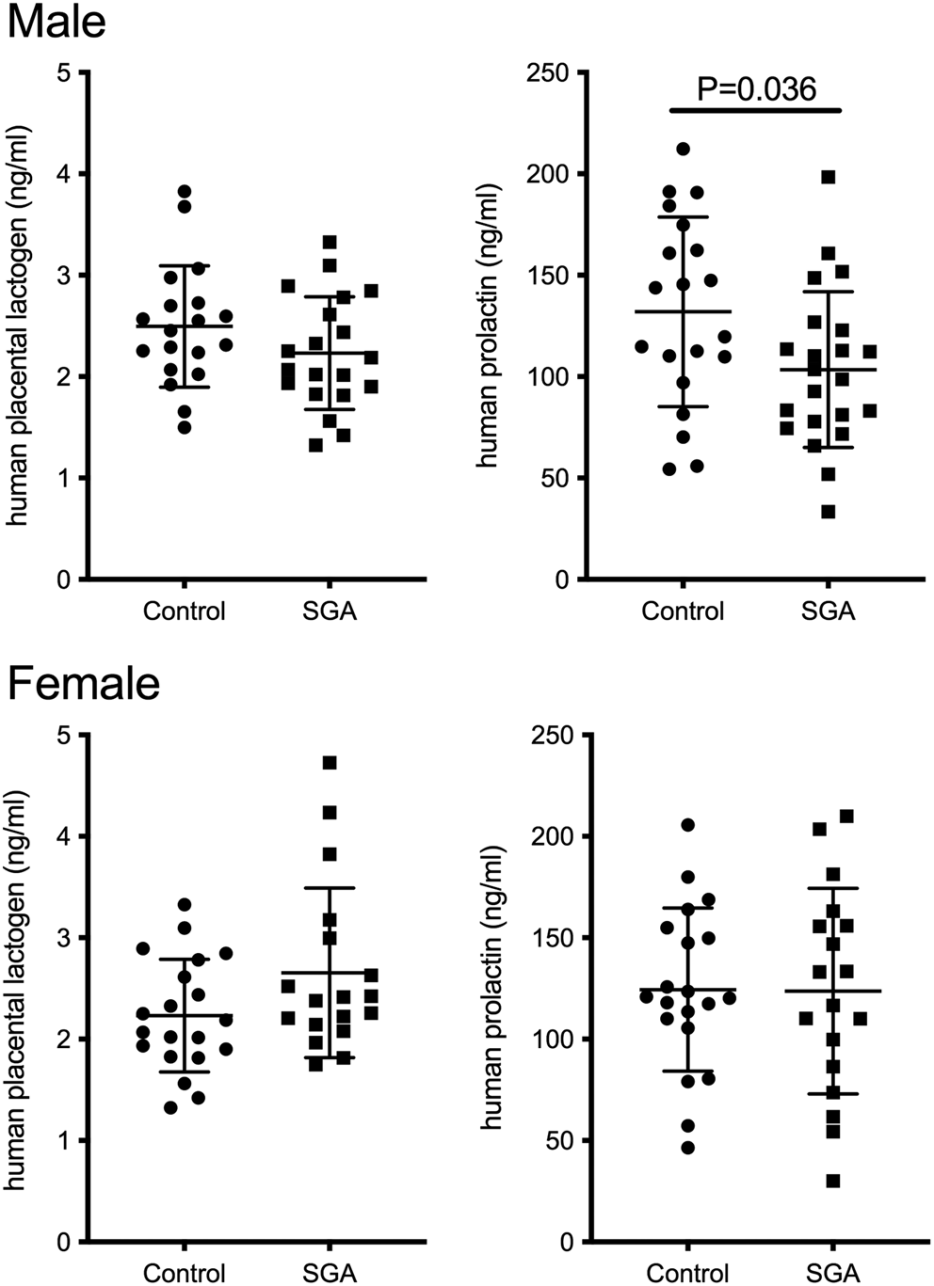
Placental lactogen (hPL) and prolactin concentrations in plasma samples from pregnant women carrying boys (*top*) or girls (*bottom*). Plasma samples were collected at 20 weeks of gestation from women who went on to have babies of normal birth weight (control, boys: *n*  = 20, girls: *n*  = 20) or SGA, boys: *n*  = 22, girls: *n*  = 18). Boys: hPL *P* = 0.164, prolactin *P* = 0.036, Student’s *t*-test, Girls: hPL *P* = 0.126, Mann–Whitney nonparametric test as data were not normally distributed (SGA group *P* < 0.05 D’Agostino and Pearson normality test)), prolactin *P* = 0.959, Student’s *t*-test. Data shown as mean ± s.d.

No differences were seen at week 20 of gestation between the uncomplicated pregnancy and SGA groups in maternal hPL and prolactin concentrations when the fetus was female (placental lactogen: *P* = 0.1258 two-tailed Mann–Whitney tests, prolactin: *P* = 0.9586 two-tailed *t*-test) (Fig. 3). When the fetus was male, maternal prolactin concentrations were significantly lower at 20 weeks of gestation in SGA pregnancies compared to uncomplicated pregnancies (prolactin: *P* = 0.0361 two-tailed *t*-test) while maternal hPL was similar in both groups (placental lactogen: *P* = 0.1749 two-tailed Mann–Whitney tests) (Fig. 3).

## Discussion

In current clinical practices less than 50% of pregnancies with SGA infants are usually detected before birth. Early detection of SGA pregnancies and timely delivery improves outcomes and is ,therefore, an important research priority. The aim of the present study was to determine whether either prolactin or placental lactogen might serve as a useful biomarker to help predict SGA infants.

Lactogenic hormones such as prolactin and placental lactogen are markedly increased during pregnancy, and play important roles in maternal adaptation to support the growing fetus (Augustine *et al.* 2008, Newbern & Freemark 2011). As well as mammary gland development, prolactin receptor activation during pregnancy, by either prolactin or placental lactogens, has been implicated in important roles in maternal metabolic adaptations, including induction of pregnancy-specific glucose regulation to direct glucose towards the fetus (Sorenson & Brelje 1997, Baeyens *et al.* 2016), and increasing food intake to meet the energy demands of the growing fetus and prepare for lactation (Augustine & Grattan 2008, Augustine *et al.* 2008). Prolactin action in the fetus is also involved in a range of developmental and growth functions (Winters *et al.* 1975, Hauth *et al.* 1978, Pullano *et al.* 1989). Therefore, we hypothesized that inadequate prolactin and/or placental lactogen during pregnancy may be associated with pregnancies with SGA infants.

We found a trend (*P* = 0.058) for lower prolactin concentrations at 20 weeks of gestation in pregnant women who went on to have SGA babies (<10th centile), however, this potential relationship was not apparent in more extreme SGA (<5th centile) and lends strength to the conclusion that a single measurement of prolactin at 20 weeks of gestation is not a useful biomarker of SGA. When the data was analyzed based on the sex of the fetus, we found a significant reduction in maternal prolactin concentrations in SGA pregnancies compared to uncomplicated pregnancies when the fetus was male. However, there was still quite an overlap between the prolactin concentrations in these two groups, suggesting that maternal prolactin concentrations by itself is unlikely to be a useful biomarker *per se* even in pregnancies carrying male fetuses. Whether it might contribute to a wider risk score, associated with assessment of key clinical variables (McCowan *et al.* 2013) requires further investigation. Overall, there was no correlation between plasma prolactin concentrations at 20 weeks and birth weight (data not shown). Whether plasma prolactin may be a useful biomarker of SGA at other time points during pregnancy cannot be ruled out by these data. At term, hPL has been significantly associated with infant birth weight (Janssen *et al.* 2016) and potentially this relationship would be detectable at an earlier time, thus future work investigating time points between 20 weeks and term would be warranted.

Previous work has indicated that in pregnancies carrying female fetuses, maternal hPL is lower in SGA pregnancies compared to uncomplicated controls (Lagerstrom *et al.* 1990). We did not observe this difference in hPL and the reasons for this inconsistency between previous work and the current study are unknown. Previous work was a prospective cohort study and hence their SGA group size was smaller than the current study. It should be noted, however, that the difference in group size between our female SGA group (*n* = 18) and the previous study (*n* = 11) was not large. It is also possible that the definition of SGA in this current study and that of SFD (small for date) for the previous study are not comparable given the 30 year lapse in time between studies.

Our sample size was only 40 cases and 40 controls. Based on the apparent effect size and the high variability, we observed for both hormones; a *post hoc* power analysis indicated that a larger study with at least 95 patients per group would be required to determine whether the observed trend was significant when data was not analyzed by fetal sex. Within such a larger study, analysis of clinical and other biomarker variables that could be assessed alongside prolactin concentrations may strengthen the predictive value, especially for male fetuses. Our study only assessed one timepoint, and future work should also assess the relevance of other time points in pregnancy that may be of use for aiding diagnosis of SGA.

Overall, while maternal prolactin concentrations did tend to be lower in SGA pregnancies, there was great variation and overlap of prolactin concentrations in both groups. A significant sex-specific association was observed, with SGA pregnancies carrying male fetuses having lower maternal prolactin concentrations compared to uncomplicated pregnancies carrying male fetuses. Our work suggests that neither prolactin nor placental lactogen at 20 weeks gestation are likely to be useful biomarkers for SGA, however this does not rule out their potential to be useful at a different time point of pregnancy.

## Declaration of interest

The authors declare that there is no conflict of interest that could be perceived as prejudicing the impartiality of the research reported.

## Funding

This work was funded by the New Zealand Health Research Council. This funding body had no role in the design of the study, collection, analysis, and interpretation of the data, nor in the writing of this manuscript.

## Ethics approval and consent to participate

Ethics approval was obtained from local ethics committees, including New Zealand (AKX/02/00/364) and all women provided written informed consent.

## Availability of data and material

The data sets from this manuscript is available by reasonable request from the corresponding author.

## Author contribution statement

C L, R S T, D R G, and L M E M were involved in the conceptualization of the study and the design of the work. S R L analyzed the data and wrote the manuscript. S R L, R S T, D R G, and L M E M contributed to the interpretation of the data and edited the manuscript. All authors have read and approved the manuscript.
